# Stochastic Physiological Gaze-Evoked Nystagmus With Slow Centripetal Drift During Fixational Eye Movements at Small Gaze Eccentricities

**DOI:** 10.3389/fnhum.2022.842883

**Published:** 2022-05-12

**Authors:** Makoto Ozawa, Yasuyuki Suzuki, Taishin Nomura

**Affiliations:** Department of Mechanical Science and Bioengineering, Graduate School of Engineering Science, Osaka University, Osaka, Japan

**Keywords:** physiological gaze-evoked nystagmus, fixational eye movements, ocular drift, centripetal drift, microsaccade, inter-microsaccadic interval, small gaze eccentricity

## Abstract

Involuntary eye movement during gaze (GZ) fixation, referred to as fixational eye movement (FEM), consists of two types of components: a Brownian motion like component called drifts-tremor (DRT) and a ballistic component called microsaccade (MS) with a mean saccadic amplitude of about 0.3° and a mean inter-MS interval of about 0.5 s. During GZ fixation in healthy people in an eccentric position, typically with an eccentricity more than 30°, eyes exhibit oscillatory movements alternating between centripetal drift and centrifugal saccade with a mean saccadic amplitude of about 1° and a period in the range of 0.5–1.0 s, which has been known as the physiological gaze-evoked nystagmus (GEN). Here, we designed a simple experimental paradigm of GZ fixation on a target shifted horizontally from the front-facing position with fewer eccentricities. We found a clear tendency of centripetal DRT and centrifugal MS as in GEN, but with more stochasticity and with slower drift velocity compared to GEN, even during FEM at GZ positions with small eccentricities. Our results showed that the target shift-dependent balance between DRT and MS achieves the GZ bounded around each of the given targets. In other words, GZ relaxes slowly with the centripetal DRT toward the front-facing position during inter-MS intervals, as if there always exists a quasi-stable equilibrium posture in the front-facing position, and MS actions pull GZ intermittently back to the target position in the opposite direction to DRT.

## Introduction

Human eyes always keep moving, even when one tries to fixate gaze (GZ) steadily. In this way, eyeball posture and the resultant GZ position fluctuate mostly involuntarily with small amplitudes during GZ fixation, referred to as fixational eye movement (FEM) (Ditchburn and Ginsborg, 1953; Rucci and Poletti, 2015), although recent studies report the voluntary aspects of FEM (Willeke et al., 2019). The corresponding time series of GZ position is referred to here as the GZ time series. It has been known that FEM contributes to visual perceptual constancy by counteracting sensory adaptation (Martinez-Conde et al., 2004). The GZ time series during FEM is composed of two types of eye movements: the drifts-tremor (DRT) and the microsaccade (MS). DRT is a slowly migrating quasi-continuous eye movement, like a Brownian motion, whose spectral power is dominated at a low-frequency band below 40 Hz, with the remaining power widely distributed in the range less than 100 Hz (Findlay, 1971; Ezenman et al., 1985). On the other hand, MS is a ballistic eye movement with a mean saccadic amplitude of about 0.3° during FEM (Martinez-Conde et al., 2009), which occurs approximately two times per second with Poisson-point-process-like inter-MS intervals (Cunitz and Steinman, 1969; Engbert and Kliegl, 2004; Engbert and Mergenthaler, 2006; Otero-Millan et al., 2008).

When the GZ target is located at an eccentric position, typically with an eccentricity more than 30°, which forces one eyeball to rotate largely away from the nose and the other eyeball to rotate toward the nose, the GZ position, defined as the binocular eye position, exhibits oscillatory movements alternating between a centripetal drift toward the front-face position and a centrifugal saccade back to the fixation target position, with a mean saccadic amplitude of about 1° and the oscillation period in the range of 0.5–1.0 s. This phenomenon is known as the physiological gaze-evoked nystagmus (physiological GEN), also called end-point nystagmus (EPN). Recent studies suggested that healthy people commonly exhibit physiological GEN at wide horizontal angles of the fixation target, from −40° to 40° across the entire range that humans can GZ (Whyte et al., 2010; Bertolini et al., 2013). On the other hand, dysfunctions of the brainstem or cerebellum can cause GZ instability, leading to pathological GEN (Cannon and Robinson, 1987; Versino et al., 1996). In both pathological and physiological GEN, the incidence rate of GEN and the absolute velocity of centripetal drifts increases with increasing GZ angle, i.e., high GZ eccentricity (Eizenman et al., 1990; Büttner and Grundei, 1995; Leigh and Zee, 2015; Tarnutzer et al., 2015). These studies imply that pathological GEN and physiological GEN share common neural networks within the cerebellum and the brainstem that has an oculomotor velocity-to-position neural integrator with leakiness. For example, increasing the amount of alcohol intake that impairs cerebellar oculomotor functions makes centripetal drifts faster with increased GZ eccentricity (Romano et al., 2017).

The relationships between FEM and physiological GEN have not been clearly understood. Bertolini et al. (2013) reported a linear relationship between a horizontal centripetal drift velocity of and horizontal GZ eccentricity (GZ angle of the fixation target) in healthy people, in which the slope of the linear relationship was about 0.02 [°/s]/[°], i.e., the horizontal centripetal drift velocity increases by the amount of 0.02/s per 1 of increase in the horizontal GZ eccentricity, for small GZ eccentricities less than 20°, and the slope was altered to about 0.05 [°/s]/[°] for large GZ eccentricities in the range of 20° to 40°. Their estimates imply a centripetal drift velocity of 0.2°/s at a horizontal fixation point of 10°, which is non-negligibly large. To establish a GZ fixation with the error caused by such a non-negligible centripetal DRT velocity, the centrifugal MS must work for counterbalancing. However, because the fixation target used by Bertolini et al. was an LED that flashed briefly for 0.050 s every 2 s and moving between −40° and 40° synchronously with the flashing, the FEM data acquired for each fixation point was too short to characterize the relationships between centripetal DRT and centrifugal MS in FEM in healthy people.

The purpose of this study was to characterize centripetal DRT and centrifugal MS more accurately than previous studies as a function of the horizontal position with small GZ eccentricities, and to elucidate the counterbalanced coordination between centripetal DRT drift and centrifugal MS jumps. To this end, we measured FEM in young healthy participants to acquire the corresponding GZ time series for a small visual target presented in front of the face, and investigated how their characteristics change depending on the horizontal position of the target, particularly in the horizontal component of FEM. In the data analysis, we decomposed the GZ time series into DRT components and MS components to define the DRT time series consisting of DRT components only, as well as MS time series consisting of MS components only. Based on the DRT and MS time series, we investigated how they could be characterized differently depending on horizontal GZ position.

## Materials and Methods

### Measurements of FEM

#### Subjects

Eight healthy young adults (mean age 23.4, ranging from 22 to 25 years, all men) participated in the experiment. All subjects had normal or corrected to normal vision. They received monetary rewards (4,800 Japanese Yen) for their participation. The experiment was approved by the local ethics committee of Osaka University and was conducted under the Declaration of Helsinki. All participants gave written informed consent.

#### Experimental Systems

Binocular eye positions were obtained using a fast video-based eye movement monitor system (a dark pupil eye tracking system; iView X Hi-Speed, SensoMotoric Instruments, Teltow, Germany) with a sampling frequency of 500 Hz. A standard 13-point calibration was performed to align the eye and computer display coordinate systems. Subjects were seated in a chair in a black room, and their head was supported on a chin/forehead rest that included the support for the camera for eye tracking. A bite bar was also used for additional support for head fixation. A computer screen with a refresh rate of 120 Hz and an area of 1, 920 pixels × 1, 080 pixels for 52.4 cm × 29.5 cm width and height was placed 72.1 cm from the eyes. The display was filled with a uniform white background of constant illumination (42.1 lux), regardless of the appearance and disappearance of the small visual target used in the task described below.

#### Experimental Protocol

**Figure 1:**
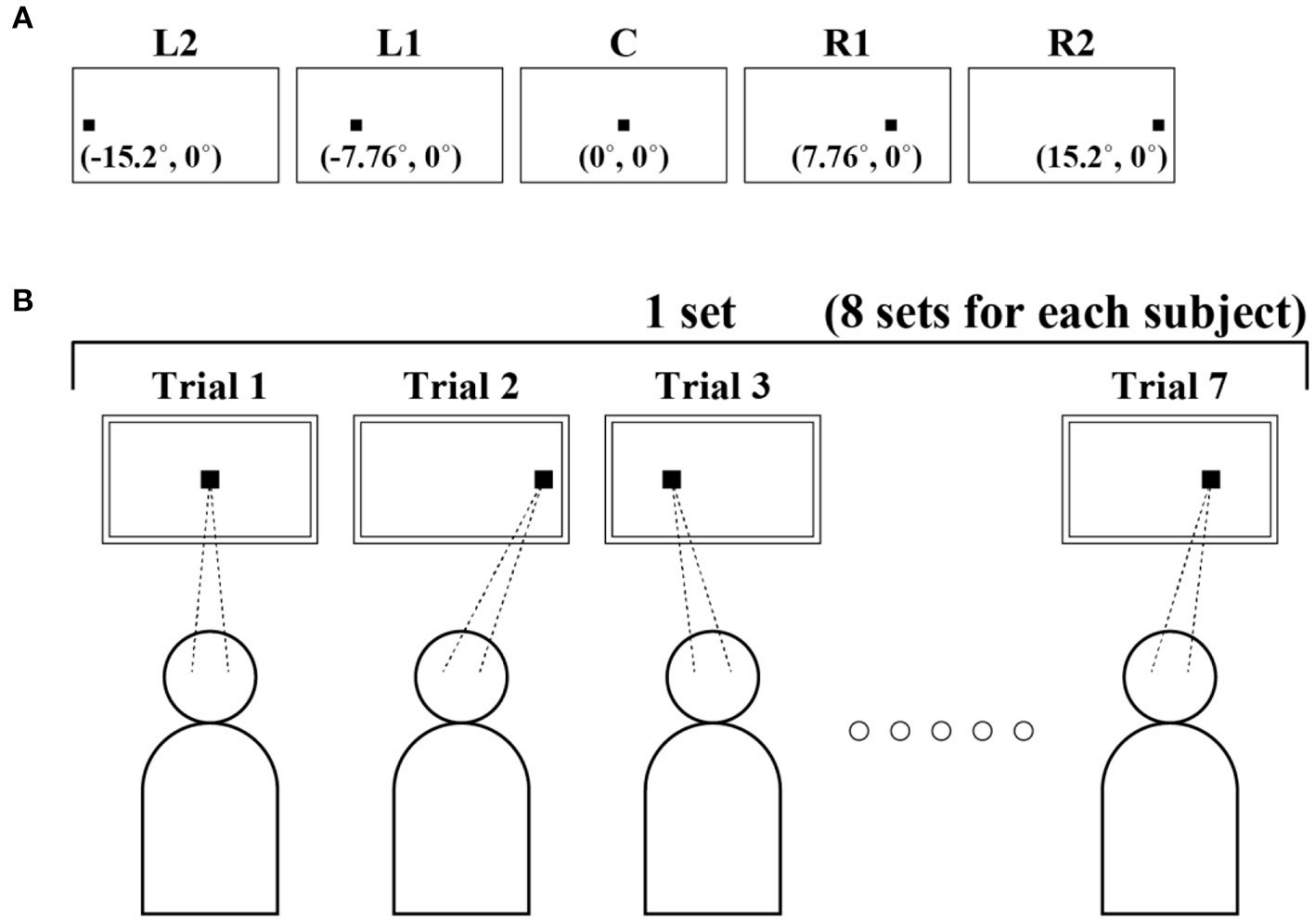
Schematic diagram of the experiment. **(A)** Five target positions from the left to right, which were referred to as L2, L1, C, R1, and R2, respectively. **(B)** An experimental protocol. A small target was projected at 0 in the vertical direction and at −15.2°, −7.76°, 0°, 7.76°, and 15.2° in the horizontal direction. Fixational eye movement (FEM) was measured to obtain gaze (GZ) time series for each of these five positions of the fixation target. Single trials lasted 35 s, which were interrupted by several eye blinks. The target position for each trial was chosen pseudo-randomly from the five locations. Each subject performed 56 trials in total.

To investigate the dependence of FEM on horizontal GZ direction, we prepared a small fixation target on the display at five locations that differed only in the horizontal direction (Figure 1A). The target was persistently located at 0 in the vertical direction, and at −15.2°, −7.76°, 0°, 7.76°, and 15.2° in the horizontal direction. Five target locations (conditions) from the left to right were referred to as L2, L1, C, R1, and R2, respectively. We measured FEM to acquire the corresponding GZ time series for those five locations of the fixation target. The fixation target was a small black square with a size of 7 pixels × 7 pixels (0.15°). The target location was randomly chosen and presented to each subject. Subjects were informed that the fixation target appeared somewhere in the horizontal position at the vertical center of the display, and were asked to fixate on the target while it appeared as accurately as possible. Subjects were allowed to blink freely during the fixation task. One session was composed of seven fixation trials. Each single trial lasted 35 s, which was interrupted by several eye blinks, for a fixed target position that was chosen pseudorandomly from the five positions. Each subject performed eight sessions, i.e., 56 trials in total on a single experimental day. A pseudorandom sequence of 56 target positions used in 56 trials for the eight sessions was predetermined, in which the randomness was manipulated so that each of the four positions was chosen 11 times and the remaining one position was chosen 12 times. We provided a break of 10 s between trials and of 10–30 min rest between sessions. Data acquisition for each subject lasted for ~4 h. The schematic view of the experimental protocol is shown in Figure 1B.

### Data Analysis

#### Preprocessing

The first and last 2.5-s segments of the GZ time series of each trial were removed to avoid possible effects of the initial transient and the expectation of the end of the trial. Blinks were detected by referring to the known properties of blinks (Caffier et al., 2003), automatically for complete blinks and manually with visual inspections for incomplete blinks. For automatic detection, the onset and offset of each blink were detected based on the vertical pupil diameter, where a value of 2/3 of the mean diameter of each GZ series was used as the threshold for detecting the onset and offset of each blink. We removed a 1.0-s long segment of the GZ time series, as a blink segment, from the pre-blink time of 0.22 s (±0.074 s) before onset to the post-blink time of 0.53 s (±0.18 s) after offset. Pre-blink and post-blink intervals were determined such that the sum of pre-blink, post-blink, and blink intervals was 1.0 s, and the ratio between pre-blink and post-blink intervals was 1:2.4. By removing the data segments during the detected blinks, the GZ series of each trial was divided into short segments corresponding to the inter-blink intervals. Each of the short segments obtained represents a sample path of the GZ time series. We excluded data segments (typically corresponding to very short inter-blink interval cases) that did not contain any MS events that were identified by the method described below. In this study, we focused on the binocular aspects of FEM only in the horizontal direction. Thus, we computed the average time series of the left and right GZ positions in the horizontal direction for further analysis. Note that, the version components are emphasized through the averaging of the left and right GZ time series, because the vergence components cancel and would be almost absent from the averaged binocular GZ time series. Note also that the version position was defined as the average between the left and right eye positions, as in previous studies (Collewijn et al., 1997; Quinet et al., 2020). Because the center of the GZ direction is not decided by the dominant eye, but is located between the two eyes as the cyclopean eye (Ono and Barbeito, 1982), the average binocular GZ values approximate the center of the GZ direction. In this way, any GZ time series hereafter for further analysis was redefined as the binocular GZ time series averaged for an inter-blink interval.

#### Decomposing GZ Time Series Into DRT and MS Series

**Figure 2:**
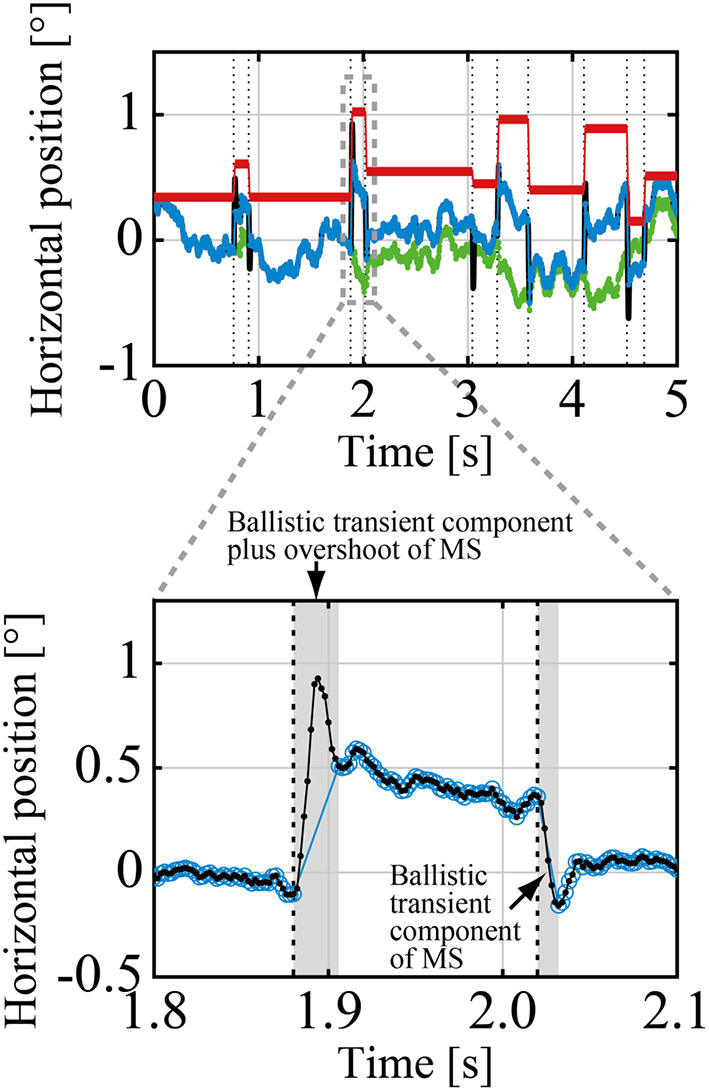
A representative example of the raw-GZ time series *X*_*rGZ*_(*t*) (black curve, mostly behind the blue curve), the GZ time series *X*_*GZ*_(*t*) (blue curve), which was decomposed into the drifts-tremor (DRT) time series *X*_*DRT*_(*t*) (green curve), and the microsaccade (MS) time series *X*_*MS*_(*t*) (red line). The vertical dotted lines indicate the time instances when MS events (MS onset and offset) occurred. The enlarged view compares the details of the raw-GZ time series *X*_*rGZ*_(*t*) (small black filled circles) with the transient and overshoot ballistic components of two MS events and the GZ time series *X*_*GZ*_(*t*) (blue open circles). The transient and overshoot ballistic components were eliminated from the raw GZ to obtain the GZ time series.

A GZ time series can be decomposed into DRT components and MS components using an algorithm summarized here (see the Supplementary Material for a detailed mathematical description of the algorithm). After decomposition, we analyzed DRT and MS components of the GZ time series separately. To this end, we defined the DRT time series *X*_*DRT*_(*t*) and the MS time series *X*_*MS*_(*t*) consisting of only DRT and MS components, respectively, for a given GZ time series that was referred to as the raw gaze (raw-GZ) time series *X*_*rGZ*_(*t*), where *t* represents the discrete time of the data with a sampling time interval of δ_*s*_ = 0.002 s. Figure 2 shows a representative example of the experimental time series *X*_*DRT*_(*t*) and *X*_*MS*_(*t*) with the corresponding *X*_*rGZ*_(*t*). The origins (0) of those time series represented the front-facing position of the GZ, and the right and left directions for the subject were defined as the positive and negative values of the GZ positions. We defined the MS time series *X*_*MS*_(*t*) by eliminating overshoot components as well as a ballistic transient component of each MS from the raw-MS time series (Ozawa and Nomura, 2019), and analyzed it for making relationships between DRT and MS clear, because we were not interested in the detailed temporal structure of each MS waveform. Note that an overshoot is a small overrun component that is followed by a small return in the opposite direction to MS immediately after MS, after which the GZ position settles at the beginning of subsequent DRT motion. In other words, the MS time series *X*_*MS*_(*t*) was obtained by replacing each transient ballistic component plus overshoot of MS in the raw-MS time series by an instantaneous jump in amplitude determined by the onset and offset of MS from the raw-MS time series [see Equation (3)]. Using *X*_*MS*_(*t*), we defined the simplified GZ time series *X*_*GZ*_(*t*), referred to simply as the GZ time series in this sequel.

In this study, we would show that each of *X*_*DRT*_(*t*) and *X*_*MS*_(*t*) exhibited a unidirectional linear trend that diffuses in the opposite direction to each other, and the slopes of their linear trend altered depending on the degree of small eccentricity of the horizontal fixation position, i.e., L2, L1, C, R1, and R2. To this end, we characterized such trends in *X*_*DRT*_(*t*) and *X*_*MS*_(*t*), and the variation of *X*_*GZ*_(*t*) as the sum of those two types of time series. In particular, we quantified the slopes of the linear trend of *X*_*DRT*_(*t*) and *X*_*MS*_(*t*) by the least-squares linear regression of *X*_*DRT*_(*t*) and *X*_*MS*_(*t*), denoted by μ_*DRT, k*_ and μ_*MS, k*_, respectively, for the *k*th sample path. Then, subject-wise mean values of the slopes, denoted ${\hat{\mu }}_{DRT}$ and ${\hat{\mu }}_{MS}$, were computed for each target position. The mean slope values across the eight subjects were summarized as boxplots for L2, L1, C, R1, and R2 to elucidate how the ${\hat{\mu }}_{DRT}$ and ${\hat{\mu }}_{MS}$ altered depending on the degree of eccentricity of the horizontal fixation point.

#### Random-Walk Analysis of the GZ and DRT Time Series

The diffusion properties of *X*_*DRT*_(*t*) and *X*_*GZ*_(*t*) were characterized by the mean squared displacements (MSD) as functions of the time lag τ · δ_*s*_ defined as follows:


(1) $$\begin{array}{r} \langle {\left(\Delta x_{GZ}\left(\tau \right)\right)}^{2}\rangle =\frac{1}{N-\tau }\overset{N-\tau }{\underset{j=1}{\sum }}|x_{GZ}\left(j+\tau \right)-x_{GZ}\left(j\right){|}^{2}, \end{array}$$

(2) $$\begin{array}{r} \langle {\left(\Delta x_{DRT}\left(\tau \right)\right)}^{2}\rangle =\frac{1}{N-\tau }\overset{N-\tau }{\underset{j=1}{\sum }}|x_{DRT}\left(j+\tau \right)-x_{DRT}\left(j\right){|}^{2}, \end{array}$$

where *N* is the total number of data included in the time interval of *T*_*GZ*_ for a given sample path. Note that MSD is also known as the stabilogram diffusion analysis (Collins and De Luca, 1993; Engbert and Kliegl, 2004). Double-log plots of MSD as a function of τ for each target position were obtained by performing an ensemble average over all sample paths for each subject. From the estimated MSD scaling exponent, we characterized the degree of diffusion of *X*_*DRT*_(*t*) and *X*_*GZ*_(*t*) in comparison with the Brownian motion that exhibits the unit scaling exponent (which is equal to 2*H* for the Hurst exponent *H*) with its variance determined as the averaged variance of the increment per second of the DRT time series.

#### Detection and Simplification of MS

**Figure 3:**
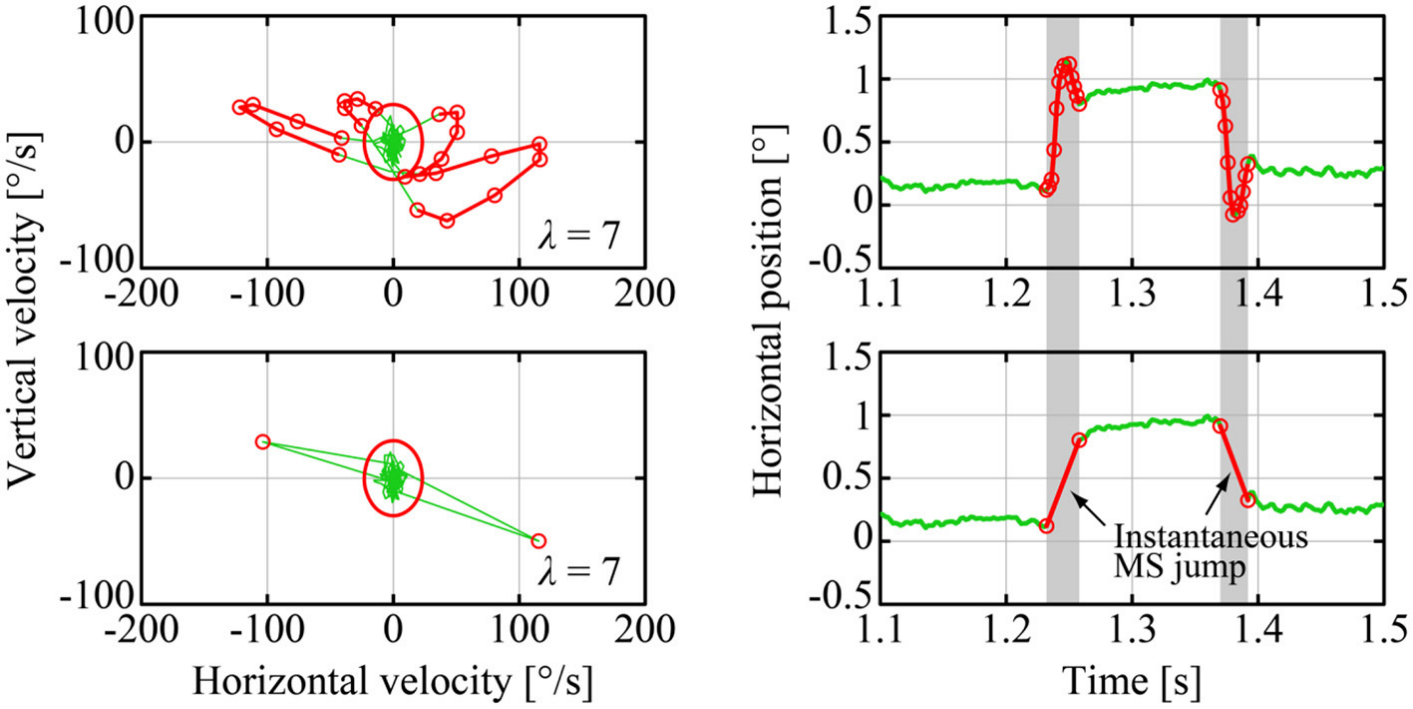
Detection and simplification of MS events. The upper panels show an example of raw-GZ data with an MS event detected by the Engbert and Kliegl (E&K) method in the *v*_*x*_ − *v*_*y*_ plane (left panel) and the corresponding time profile (right panel), where the red curves are MS components and the green curves are DRT components. The lower panels are for the corresponding GZ data, in which MS events were simplified, referred to simply as MS in this paper, by eliminating the transient and overshoot ballistic components from the raw-MS waveforms for further analysis performed in this paper.

We detected MS events using the method of Engbert and Kliegl, referred to as the E&K method (Engbert, 2006) as illustrated in Figure 3. In the E&K method, a parameter λ was introduced to determine the velocity ellipse size in the *v*_*x*_-*v*_*y*_ plane, where *v*_*x*_ and *v*_*y*_ were the velocities of the GZ motion in the horizontal and vertical directions, respectively [see Engbert and Kliegl (Engbert, 2006) for the definition of the parameter λ]. In this study, we used λ = 7 for all target position conditions. Some MS events acquired from eye movements measured by a pupil-based eye tracking system and detected by the E&K method might contain overestimated overshoot components (Otero-Millan et al., 2014; Nyström et al., 2016; Ozawa and Nomura, 2019). The validity of the MS events detected by the E&K method in the current study was confirmed by drawing the main sequence diagrams for the MS events (see Supplementary Figure 1).

As mentioned above, the MS time series *X*_*MS*_(*t*) for our analysis were obtained by replacing each transient trajectory plus the overshoot component of MS in the raw-MS time series *X*_*rMS*_(*t*) with an instantaneous jump (see Figures 2, 3). More specifically, for an onset time of the *i*th MS of the *k*th sample path of the raw-MS series, denoted by *X*_*rMS, k*_(*t*), occurred at $t_{on}^{i}$ and the corresponding offset time at $t_{off}^{i}$, the amplitude of the *i*th MS (size of the instantaneous jump) was defined as


(3) $$\begin{array}{r} w_{i,k}=X_{rMS,k}\left(t_{off}^{i}\right)-X_{rMS,k}\left(t_{on}^{i}\right) \end{array}$$

where $t_{on}^{i}\cdot \delta_{s}$ and $t_{off}^{i}\cdot \delta_{s}$ were the time instances when GZ velocity trajectory in the *v*_*x*_-*v*_*y*_ plane cut the velocity ellipse of the E&K method from inside to outside and from outside to inside, respectively. Note that, in *X*_*MS*_(*t*), any MS occurs in unit time step, because transient plus overshoot portions of the corresponding MS in *X*_*rMS*_(*t*) were eliminated by the definition of *X*_*MS*_(*t*). Positive and negative amplitudes *w*_*i, k*_ for the *i*th MS in the *k*th sampling path means that the corresponding MS jumps to the right and left, respectively, and their absolute value represents the jump size. The rightward and leftward MS events in the *k*th sampling path were counted separately when necessary, by which the signed amplitude of the *i*th rightward MS and the *j*th leftward MS were denoted, respectively, by *w*_+, *i, k*_, *w*_−, *j, k*_. The total number of rightward and leftward MS events in the *k*th sampling path were denoted by *n*_+, *k*_ and *n*_−, *k*_, respectively. The subject-wise means of *w*_*i, k*_, regardless of the jump direction, as well as the subject-wise means of *w*_+, *i, k*_ and *w*_−, *j, k*_, across all sampling paths were computed and denoted by ŵ, ŵ_+_, and ŵ_−_, respectively.

#### Temporal Structure of MS Time Series

The temporal structure of the MS time series was characterized by the histogram of the inter-microsaccadic intervals (IMSI) in the right and left directions. Because the probability of the occurrence of MS events has been approximated by a Poisson-point-process (Engbert, 2006), for which the IMSI obeys the exponential distribution with the exponential probability density function (PDF), we approximated the histograms of IMSI in each of the rightward (+) and leftward (–) directions by


(4) $$\begin{array}{r} p\left(x;\lambda_{\pm }\right)=\lambda_{\pm }e^{-\lambda_{\pm }x} \end{array}$$

for IMSI values *x* = IMSI_+_ between subsequent rightward MS events with occurrence frequency λ_+_ and for *x* = IMSI_−_ between subsequent leftward MS events with occurrence frequency λ_−_. For the *k*th sample path of *X*_*GZ*_(*t*) with the data length (the inter-blink interval) *T*_*GZ, k*_, the estimates of the Poisson rates λ_+_ and λ_−_, denoted, respectively, by Λ_+, *k*_ and Λ_−, *k*_, were obtained as the inverse of the mean MS interval, i.e.,


(5) $$\begin{array}{r} \Lambda_{+,k}=\frac{n_{+,k}}{T_{GZ,k}},\;\Lambda_{-,k}=\frac{n_{-,k}}{T_{GZ,k}}, \end{array}$$

where *n*_+, *k*_ and *n*_−, *k*_ were the numbers of rightward and leftward MS events, respectively. Moreover, we also computed the occurrence frequency of the total number of MS events, regardless of the direction of the jumps, as


(6) $$\begin{array}{r} \Lambda_{k}=\frac{n_{+,k}+n_{-,k}}{T_{GZ,k}}, \end{array}$$

for the *k*th sample path. For those MS rate parameters, the subject-wise mean values, i.e., ${\hat{\Lambda }}_{+}$, ${\hat{\Lambda }}_{-}$, and $\hat{\Lambda }$, were computed. In addition to those estimates of MS occurrence, the slopes of the least-squares linear regression lines for semi-log plots of the IMSI_+_ and IMSI_−_ histograms were performed, by which alternative estimates of the Poisson rates for each subject, denoted by ${\hat{\lambda }}_{+}$ and ${\hat{\lambda }}_{-}$, respectively, were obtained, which should be consistent with the ${\hat{\Lambda }}_{+}$ and ${\hat{\Lambda }}_{-}$ obtained by Equation (5), if the occurrence of each of the rightward and the leftward MS events obeyed the Poisson-point-process. The consistency between ${\hat{\Lambda }}_{\pm }$ and ${\hat{\lambda }}_{\pm }$ confirms the assumption of the Poisson-point-process property of the MS events.

As described above, the unidirectional linear trend of the MS time series *X*_*MS*_(*t*) for the *k*th sample path was quantified by the slope μ_*MS, k*_ of the least-squares regression line. We computed the subject-wise mean values of μ_*MS, k*_, denoted by ${\hat{\mu }}_{MS}$. Alternatively, the linear trend of the stair-like MS time series for the *k*th sample path could be quantified using the mean jump sizes ŵ_+, *k*_>0 and ŵ_−, *k*_ <0 and their mean occurrence frequency per second by introducing the parameter *s*_*k*_ defined as follows:


(7) $$\begin{array}{r} s_{k}≜w_{+,k}\lambda_{+,k}+w_{-,k}\lambda_{-,k}. \end{array}$$

Then, ŝ as the subject-wise mean of *s*_*k*_ across all trials for each subject was also computed. Comparisons between those two types of quantifications, i.e., ${\hat{\mu }}_{MS}$ and ŝ across subjects were made to elucidate the underlying mechanisms of the unidirectional linear MS trend as well as the DRT time series. Specifically, to elucidate the dominant contributing parameters, among ${\hat{\lambda }}_{+},\;{\hat{\lambda }}_{-},\;{ŵ}_{+},$ and ŵ_−_, that characterize the temporal structure of the MS time series to the unidirectional linear trend of the MS time series, we compared the means of those four parameters across all subjects using boxplots of the parameters for the five fixation conditions, i.e., L2, L1, C, R1, and R2. Furthermore, the boxplots of the values of ŝ across all subjects for the five fixation conditions were examined, which were compared with the boxplots of the slope ${\hat{\mu }}_{MS}$ of the least-squares linear regression line, to show that an appropriately biased balance of the rates and amplitudes between rightward and leftward MS events was the major cause of the unidirectional linear trend of the MS time series. We also performed comparisons between ${\hat{\lambda }}_{+}\;$and ${\hat{\lambda }}_{-}$ and between ŵ_+_ and ŵ_−_ for each of the five target positions to examine the differences between rightward and leftward MS jumps.

#### Relationships Between the Onset Position of MS and Its Amplitude With the Direction of MS

The relationships between the onset position of MS and its amplitude were analyzed by examining their joint PDF, for which we calculated the corresponding two-dimensional histograms of the onset position and amplitude of MS with 0.25° and 0.20° bin widths, respectively. We then performed cubic spline interpolation of the histogram between the bins (meshes), which was displayed as a color map of the joint frequency of the onset positions and amplitudes of the MS events. Moreover, to investigate the relationships between the onset position and the jump direction of MS, we calculated a one-dimensional histogram with a bin width of 0.10° for each of the rightward and leftward MS events. To investigate the MS amplitude distribution, we calculated a one-dimensional histogram with a bin width of 0.10° for each of the rightward and leftward MS events. As Supplementary Material, we also calculated a one-dimensional histogram of the GZ time series for each fixation condition, corresponding to the PDF of the GZ time series, which was shown below the color map.

#### Examining the Balance Between the Linear Trends of DRT and MS

We hypothesized that the GZ fixation for each of the five target positions was established by the balance between the unidirectional linear trends of the DRT and MS time series in opposite directions. To examine such a balance, we examined scatter plots for *s*_*k*_ defied by Equation (7) against the unidirectional linear trend of DRT μ_*DRT, k*_ for all sample paths *k* across all subjects. Negative correlation with the slope of minus one in the scatter plot implies that the centripetal DRT and the centrifugal MS are completely balanced, as in the following equation


(8) $$\begin{array}{r} \mu_{DRT}=-s. \end{array}$$

Moreover, we also computed the means of μ_*DRT, k*_ and the parameter *s*_*k*_ for all *k*th sample paths across all subjects, denoted by $\mu_{DRT}$ and *s*, respectively, for each target position, which were indicated on the scatter plots.

#### Target Position-Dependent Basic Properties of FEM

To characterize target position-dependent basic properties of FEM, other than the direct drift-related properties, we calculated statistical mean values of the following 14 metrics for the GZ time series *X*_*GZ*_(*t*), averaged across all trials (all sample paths) and all subjects for each target position: the data length *T*_*GZ*_ of *X*_*GZ*_(*t*) defined by Supplementary Equation 15 in the Supplementary Materials that represents the inter-blink interval, the time average of GZ position *X*_*GZ*_(*t*), the standard deviation (SD) of *X*_*GZ*_(*t*), the absolute error between the target position and the GZ position, the amplitude of the total MS regardless of the direction, and the rightward MS and the leftward MS (i.e., ŵ, ŵ_+_, and ŵ_−_ as defined above), the occurrence frequency $\hat{\Lambda }$ for the total MS regardless of the direction using Equation (6), and the occurrence frequencies of the rightward and leftward MS events by ${\hat{\Lambda }}_{+}$ and ${\hat{\Lambda }}_{-}$ from Equation (5) as well as by ${\hat{\lambda }}_{+}\;$and ${\hat{\lambda }}_{-}$ based on the exponential fitting of IMSI histograms, the onset position of MS relative to the mean GZ position, and the offset position of MS relative to the mean GZ position.

#### Statistical Analysis

To examine the target position dependence on any metrics characterizing the GZ, DRT, and MS time series, we performed Bartlett's test to examine the equality of variances among five target positions (L2, L1, C, R1, and R2), and confirmed it at the 5% significance level. The Tukey–Kramer test was then performed to examine the differences between them at the 5 and 1% significance level.

## Results

### Basic Statistics

Table 1 shows the dependence of horizontal GZ position on the mean values of 14 metrics averaged across all subjects. There were no significant differences between horizontal GZ positions in the following 12 metrics: mean values of the data length *T*_*GZ*_ of *X*_*GZ*_(*t*), the GZ position, the SD of the GZ position, absolute value of the error from the target, amplitude of the rightward and the leftward MS events (ŵ_+_ and ŵ_−_), occurrence frequency of the rightward and the leftward MS events (${\hat{\Lambda }}_{+}$ and ${\hat{\Lambda }}_{-}$ as well as ${\hat{\lambda }}_{+}\;$and ${\hat{\lambda }}_{-}$), occurrence frequency of the total MS events $\hat{\Lambda }$, and the relative onset position of MS. Significant differences (*p* < 0.05) were found only in the amplitude ŵ of total MS and relative MS offset position between L2 and R2. However, the SD of the GZ position and absolute error from the target tended to be large as the eccentricity was increased, although without no statistical significance.

**Table 1:** Dependence of horizontal gaze (GZ) position on the mean of each statistic for all subjects.

**Mean values across 8 subjects**	**Horizontal fixation target**				
	**L2**	**L1**	**C**	**R1**	**R2**
Data length, *T*_*GZ*_ [s]	15.6 (±8.08)	16.1 (±8.57)	16.7 (±8.78)	15.2 (±6.68)	14.5 (±8.40)
Gaze position, *X*_*GZ*_ [°]	−15.2 (±0.215)	−7.79 (±0.131)	0.0236 (±0.158)	7.77 (±0.121)	15.1 (±0.219)
SD of gaze position [°]	0.246 (±0.0406)	0.230 (±0.0250)	0.206 (±0.0152)	0.228 (±0.0354)	0.243 (±0.0271)
Absolute error from target [°]	0.363 (±0.0977)	0.299 (±0.117)	0.271 (±0.0743)	0.319 (±0.161)	0.347 (±0.0773)
Amplitude of total MS, ŵ [°] *	−0.0514 (±0.0622)	−0.0258 (±0.0459)	0.0016 (±0.0370)	0.0164 (±0.0372)	0.0392 (±0.0532)
Amplitude of leftward MS, ŵ_−_ [°]	−0.363 (±0.132)	−0.361 (±0.0996)	0.314 (±0.0875)	−0.315 (±0.103)	−0.326 (±0.105)
Amplitude of rightward MS, ŵ_+_ [°]	0.354 (±0.105)	0.348 (±0.0842)	0.310 (±0.0763)	0.329 (±0.0867)	0.354 (±0.112)
Frequency of total MS, $\hat{\Lambda }$ [1/s]	1.66 (±0.499)	1.55 (±0.478)	1.47 (±0.560)	1.57 (±0.544)	1.67 (±0.512)
Frequency of leftward MS, ${\hat{\Lambda }}_{-}$ [1/s]	0.934 (±0.274)	0.842 (±0.291)	0.745 (±0.312)	0.803 (±0.333)	0.818 (±0.287)
Frequency of rightward MS, ${\hat{\Lambda }}_{+}$ [1/s]	0.738 (±0.250)	0.727 (±0.216)	0.745 (±0.263)	0.779 (±0.206)	0.891 (±0.209)
Frequency of leftward MS, ${\hat{\lambda }}_{-}$ [1/s]	1.21 (±0.374)	1.07 (±0.325)	0.967 (±0.411)	1.09 (±0.386)	1.02 (±0.423)
Frequency of rightward MS, ${\hat{\lambda }}_{+}$ [1/s]	0.854 (±0.462)	0.966 (±0.395)	0.902 (±0.367)	1.02 (±0.335)	1.12 (±0.322)
Relative Onset position of MS to mean gaze position [°]	0.0240 (±0.0497)	0.0182 (±0.0496)	0.0044 (±0.0320)	−0.0005 (±0.0180)	−0.0052 (±0.0308)
Relative offset position of MS to mean gaze position [°] *	−0.0400 (±0.0535)	−0.0141 (±0.0463)	0.0053 (±0.0354)	0.0186 (±0.0323)	0.0420 (±0.0432)

The * symbol indicates that there was a significant difference (p < 0.05) between L2 and R2.

### GZ, DRT, and MS Time Series

**Figure 4:**
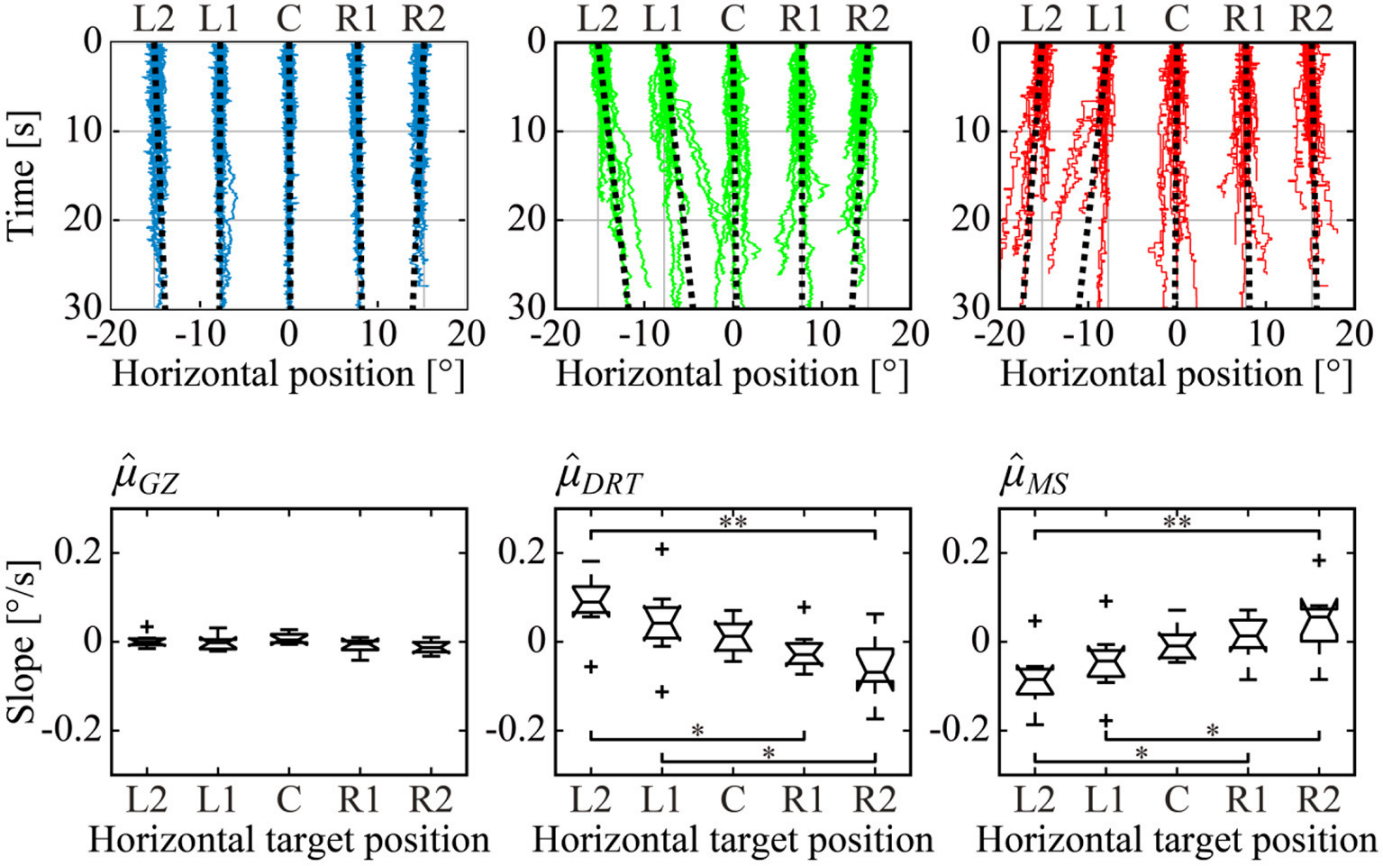
Horizontal dependence of the target position on GZ, DRT, and MS time series and the slopes on their trend. Upper panels: All sample paths of GZ (left, blue), DRT (middle, green), and MS (right, red) time series of a representative subject were superimposed for each of the five target positions. Black dotted lines are the least-squares linear regression lines, representing the linear trend of the time series for each of the five target positions. Lower panels: box plots of the mean value of the subject-wise means of slopes for each time series across all subjects. Single and double asterisks indicate that there was a significant difference between groups at the 5 and 1% significance level, respectively.

The upper panels of Figure 4 show all sample paths of the GZ time series *X*_*GZ*_(*t*), DRT time series *X*_*DRT*_(*t*), and MS time series *X*_*MS*_(*t*), for a representative subject. The least-squares linear regression line was superimposed on each of the *X*_*GZ*_(*t*), *X*_*DRT*_(*t*), and *X*_*MS*_(*t*) for each of the five fixation targets. For *X*_*GZ*_(*t*), the blue time series in the upper left panel of Figure 4, we could observe the time series fluctuating around the target position. Together with the fact that the SD of *X*_*GZ*_(*t*) and the absolute error were small, as shown in Table 1, with no dependence on target position, we confirmed that the GZ fixation was performed correctly, regardless of the horizontal target position. For *X*_*DRT*_(*t*), the green time series in the upper middle panel of Figure 4, the unidirectional linear trend toward the front-facing position (i.e., the horizontal center at 0°) was found for the target position of L2, L1, R1, and R2. That is, *X*_*DRT*_(*t*) exhibited the centripetal drift. On the other hand, in the upper right panel of Figure 4, the red time series of *X*_*MS*_(*t*) showed the unidirectional linear trend in the opposite direction to DRT, i.e., away from the front-facing position, were found for the target position of L2, L1, R1, and R2. In other words, *X*_*MS*_(*t*) exhibited a centrifugal trend. The linear trend of each time series was quantified by the slope of the least-squares linear regression line, and the mean slope averaged across all subjects for each of the five target positions as depicted in the lower panels of Figure 4. The slopes of *X*_*GZ*_(*t*) were close to 0 for all of the five target positions, and there were no significant differences in the slopes of *X*_*GZ*_(*t*) among the five target positions. That is, GZ fixation was performed correctly regardless of the fixation position. On the other hand, in the *X*_*DRT*_(*t*) and *X*_*MS*_(*t*) boxplots, the slope became steeper as the target GZ angle became larger, in the opposite direction from each other. That is, the centripetal drift of *X*_*DRT*_(*t*) and the centrifugal trend of *X*_*MS*_(*t*) became faster as the target GZ angle increased. The differences between the slopes were significant between L2 and R2 (*p* < 0.01), between L2 and R1 (*p* < 0.05), and between L1 and R2 (*p* < 0.05) for both of *X*_*DRT*_(*t*) and *X*_*MS*_(*t*). For the L2 and R2 conditions, the mean absolute values of the linear trend of *X*_*DRT*_(*t*) and *X*_*MS*_(*t*) were about 0.1°/s. Note that there were some subjects who did not exhibit the target position-dependent linear trends of *X*_*DRT*_(*t*) and *X*_*MS*_(*t*).

### Random-Walk Analysis

**Figure 5:**
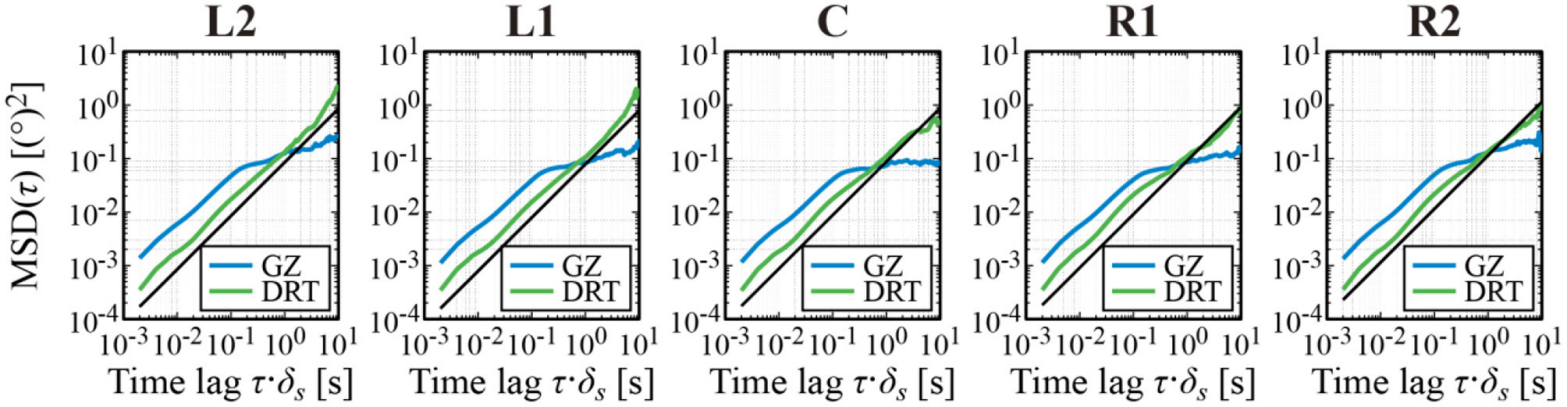
Mean square displacement (MSD) analysis of the GZ and DRT time series. The blue and green curves represent, respectively, the MSDs of the GZ and DRT time series. The black lines are the MSD of the Brownian motion for comparison. The MSD curves depicted in each panel are the ensemble average of MSD for each sample path of a representative subject.

Figure 5 shows the MSD of *X*_*GZ*_(*t*) and *X*_*DRT*_(*t*) for a representative subject for each of the five target positions. Over a wide range of time lag (0.002–10 s), regardless of the horizontal fixation condition, the scaling exponent of the DRT time series, which corresponds to 2*H* for the Hurst exponent *H*, was about 1, i.e., *H*~1/2. That is, the MSD profile of the DRT time series was similar to the MSD of the Brownian motion, regardless of the target position, despite of the fact that the slope (i.e., the velocity) of the centripetal linear trend of the DRT time series exhibited a clear dependence on the target position. This means that the centripetal DRT linear trend was not caused by a positive persistence of the DRT time series **(**see Section Discussion). On the other hand, the MSD of the GZ time series *X*_*GZ*_(*t*) exhibited a crossover phenomenon, in which the critical point (critical time lag) was located around 0.1–0.2 s. The 2H scaling exponent of *X*_*GZ*_(*t*) for the short-time scale (0.002–0.1 s) was about 1, whereas that for the long-time scale (0.2–10 s) was between 0.1 and 0.3, regardless of the target position. In summary, the MSD of *X*_*GZ*_(*t*) and *X*_*DRT*_(*t*), i.e., the random-walk property of the GZ and DRT time series did not show the dependence on the target position, despite the fact that the slope (i.e., the velocity) of the centripetal linear trend of the DRT time series and the centrifugal linear trend of the MS time series exhibited a clear dependence on the target position.

### Characterization of MS Time Series

**Figure 6:**
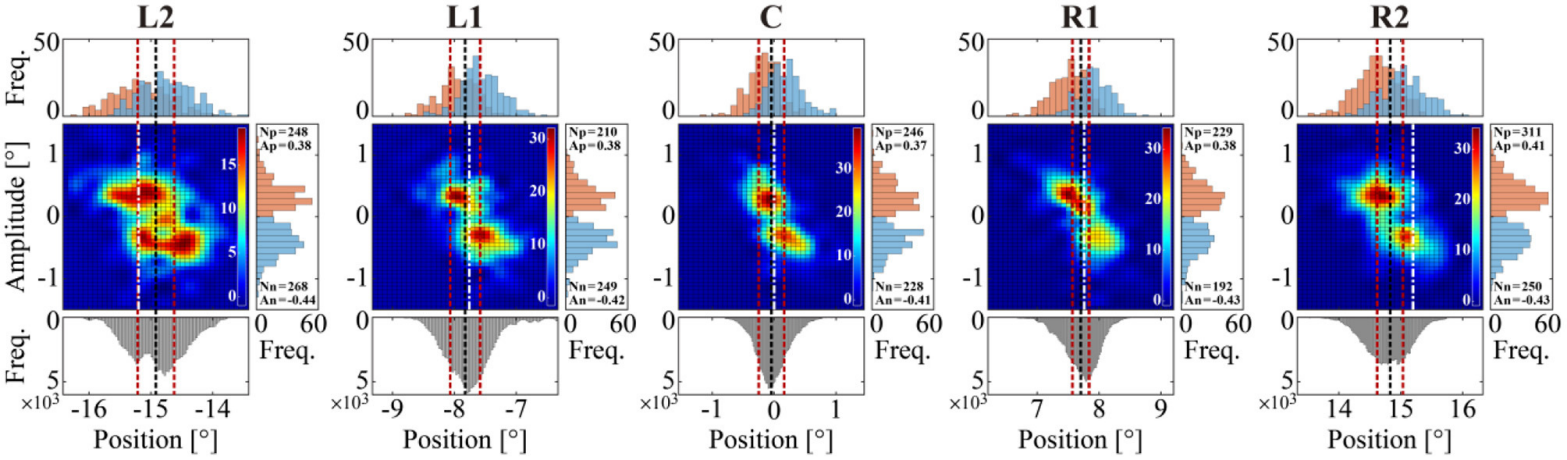
Relationships between the onset position of MS and its amplitude for each of the five target positions. Color map: two-dimensional histograms of the onset position and amplitude of MS with 0.25° and 0.20° bin widths, respectively, representing the joint frequency of onset positions and amplitudes of MS events. Upper histograms: one-dimensional histograms with a bin width of 0.10° for each of the rightward (red) and leftward (blue) MS events. Right-side histograms: one-dimensional histograms with a bin width of 0.10° for each of the rightward (red) and leftward (blue) MS events. In the right-side histograms, *N*_*p*_ and *N*_*n*_ are the rightward and leftward MS counts, respectively. Similarly, *A*_*p*_ and *A*_*n*_ are the mean amplitude of rightward and leftward MS. For each target condition, the black dotted line represents the mean value of the GZ time series, and the red dotted lines on both sides of the black line represent ±SD away from the mean value of the GZ time series. The dashed white line represents the fixation target position in each target condition.

**Figure 7:**
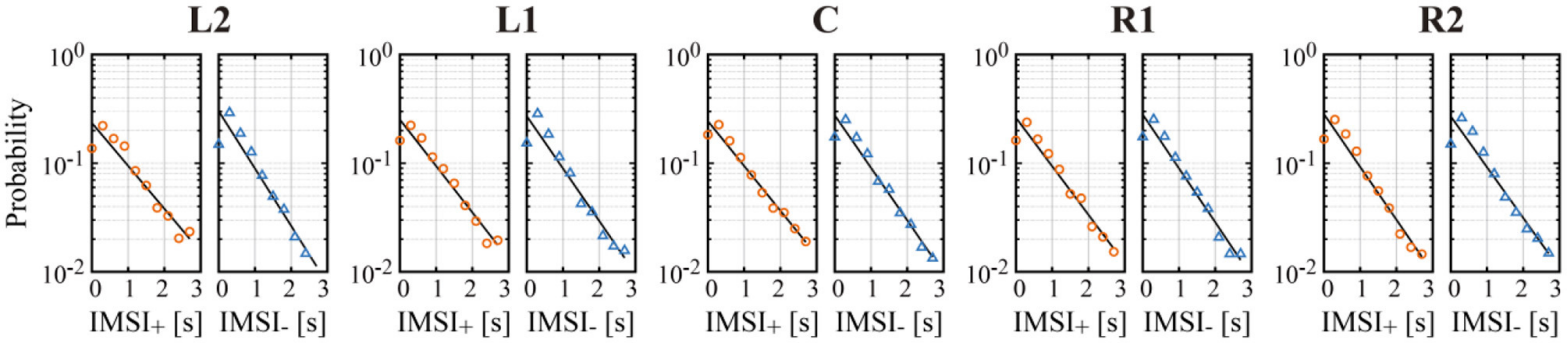
Semi-log plot of the probability of inter-microsaccadic intervals (IMSI) of all left/right MS across all subjects, with a regression line (black line) in each condition. *IMSI*_+_ plotted by the orange circles are the IMSI of rightward MS, and *IMSI*_−_ plotted by the blue triangles are the IMSI of leftward MS.

**Figure 8:**
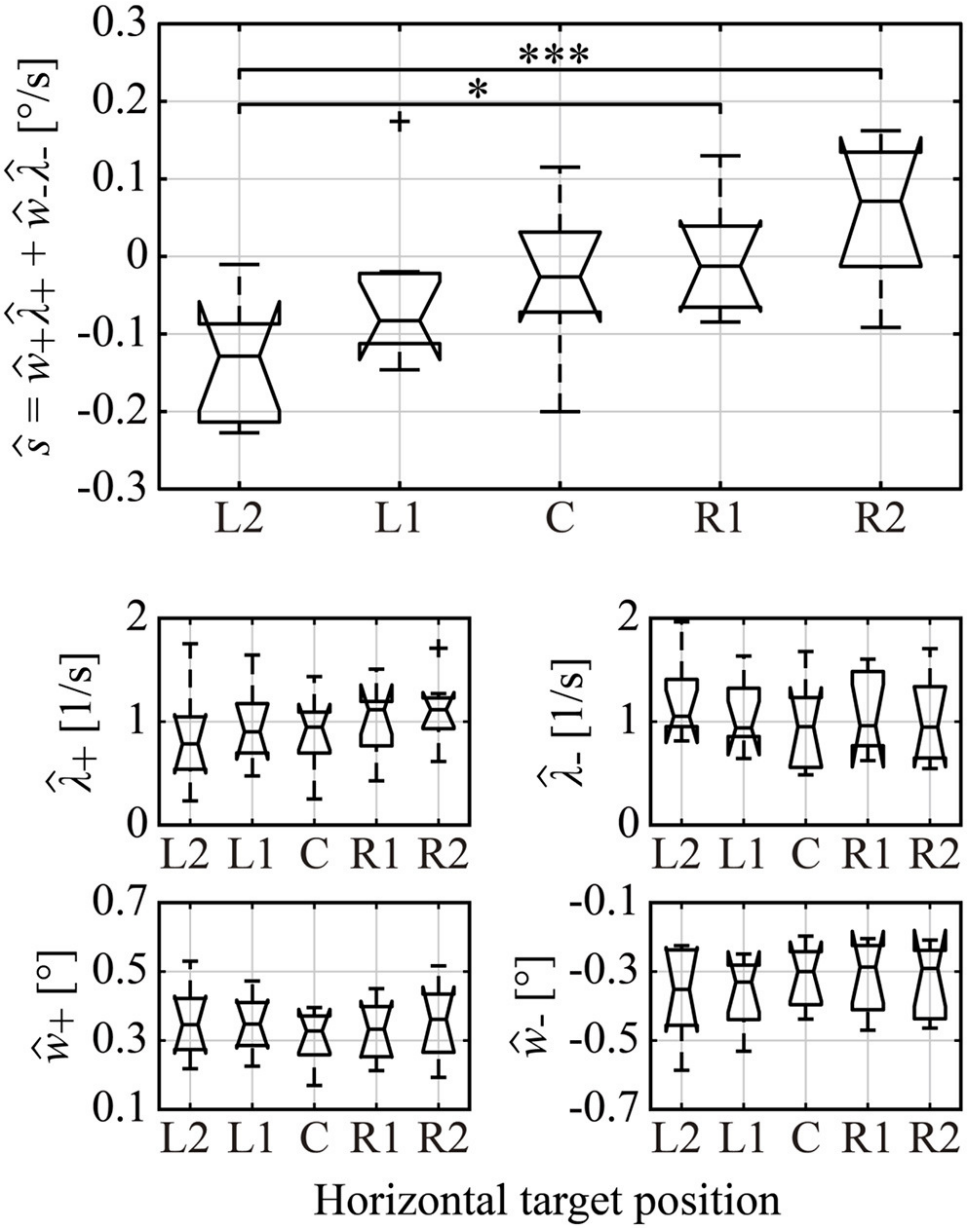
Boxplots of the estimated amplitude and occurrence frequency of right/left MS, and the total trend generated by total MS across all subjects. ŵ_+_, ŵ_−_, ${\hat{\lambda }}_{+}$, and ${\hat{\lambda }}_{-}$ represent the subject-wise mean values of the right/left MS amplitude and occurrence frequency, respectively. $ŝ≜{ŵ}_{+}{\hat{\lambda }}_{+}+{ŵ}_{-}{\hat{\lambda }}_{-}$ is the estimated total trend of MS reconstructed from ŵ_+_, ŵ_−_, ${\hat{\lambda }}_{+}$, and ${\hat{\lambda }}_{-}$. Single and triple asterisks indicate a significant difference between the groups at the 5 and 0.1% significance level, respectively.

Detailed characterizations of the MS time series are presented in Figures 6, 7 for a representative subject and in Figure 8 obtained using data from all subjects. Regardless of the target position, we found a negative correlation between MS onset position and amplitude (ŵ_+_ and ŵ_−_), as represented in each panel of Figure 6. More specifically, the onset positions were localized at locations about 0.3° from the average GZ position, in both the negative (left) and the positive (right) directions, from which MS with an amplitude of about 0.3° occurred in the positive (rightward) and the negative (leftward) directions, respectively, regardless of the target position. The histograms of MS events tended to be more horizontally stretched and became asymmetric as the target position was more distant from the front-facing position. For the target positions on the left, particularly for the L2 condition, the peaks of the histograms (in the upper side of the color maps) for the leftward MS (the blue histograms) were slightly higher than those for the rightward MS (the red histograms). Moreover, the mean value of the MS onset (the dashed vertical black line in the color map) was slightly shifted to the right from the target position (the dash-dot vertical white line). In contrast, for the target positions on the right, particularly for the R2 condition, the peaks of the rightward MS histograms (the red histograms) were slightly higher than those of the leftward MS histograms (the blue histograms). Moreover, the mean value of the MS onset (the dashed vertical black line in the color map) was slightly shifted to the left from the target position (the dash-dot vertical white line). In this way, MS events occurred more frequently toward the centrifugal direction compared to the centripetal direction. However, these differences between rightward and leftward MS events were not statistically significant, as shown in Table 1.

We confirmed in Figure 7 that the occurrence probability of IMSI with both in the positive (IMSI_+_) and negative (IMSI_−_) directions obeyed the exponential distribution with the identical rate parameter, regardless of the target position (see Table 1 for a comparison of the rates ${\hat{\lambda }}_{+}$ and ${\hat{\lambda }}_{-\;}$). Figure 8 shows the boxplots of ŝ as the mean of the subject-wise mean values of *s*_*k*_≜*w*_+, *k*_λ_+, *k*_+*w*_−, *k*_λ_−, *k*_, as well as ${\hat{\lambda }}_{+},\;{\hat{\lambda }}_{-},\;{ŵ}_{+}$ and ŵ_−_ that characterize the target position dependency of the occurrence frequency and the amplitude of MS for each of the five target positions. The top panel of Figure 8 shows that ŝ, representing the velocity of centrifugal trend of *X*_*MS*_(*t*), increased linearly from negative to positive values as the target location varied from the left to the right. This dependency was apparent and quantitatively the same as the target position dependency of the mean slope of the regression lines for the centrifugal linear tend of *X*_*MS*_(*t*) (the lower right panel of Figure 4), which counterbalanced with the opposite dependency of the mean slope of the regression line for the centripetal linear trend of *X*_*DRT*_(*t*) (the lower middle panel of Figure 4). Specifically, differences in ŝ were significant between L2 and R2 (*p* < 0.01), and between L2 and R1 (*p* < 0.05). As described for the asymmetric shape of the histograms in Figure 6, the occurrence frequencies of the rightward ${\hat{\lambda }}_{+}$ and leftward ${\hat{\lambda }}_{-}$ MS events slightly increased and decreased, respectively, as the target position varied from the left to the right (the middle row panels of Figure 8), although the pairwise comparison between those values for the five target positions were not different significantly (Table 1). We also compared the values of ${\hat{\lambda }}_{+}$ and ${\hat{\lambda }}_{-}$ for each target position, because it seemed ${\hat{\lambda }}_{+}<{\hat{\lambda }}_{-}$ for L2 and ${\hat{\lambda }}_{+}>{\hat{\lambda }}_{-}$ for R2 by the visual inspection. However, the differences between the rates of rightward and leftward MS events within the same target position were not statistically significant. Moreover, there was no recognizable nor statistically significant target position dependency in the amplitude of the leftward ŵ_−_ and rightward ŵ_+_ MS events (the middle row panels of Figure 8). In any cases, each of the parameters ${\hat{\lambda }}_{+},\;{\hat{\lambda }}_{-},\;{ŵ}_{+},$ and ŵ_−_ alone did not show significant dependency on the target position.

### Balance Between the Unidirectional Trends of the DRT and MS Time Series

**Figure 9:**
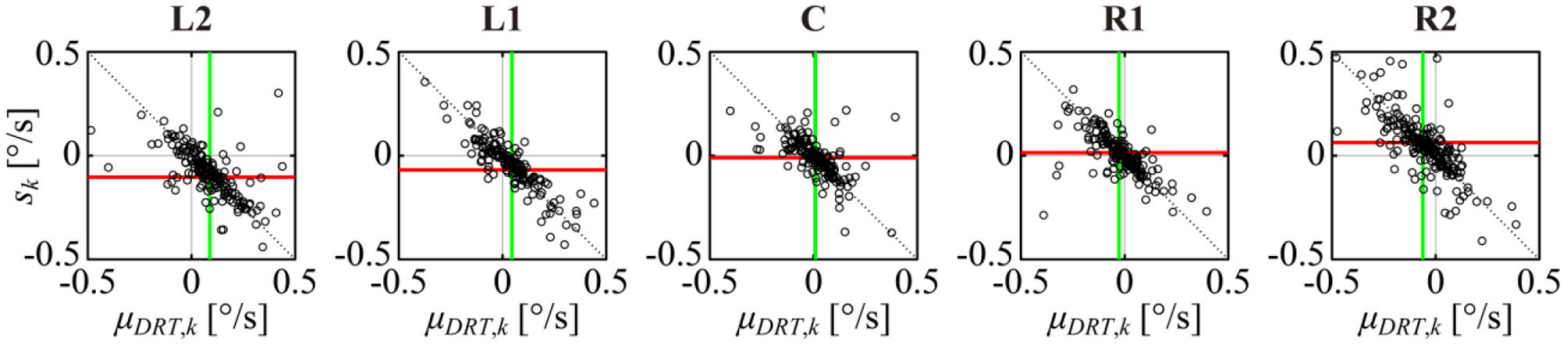
Scatter plot of a DRT trend μ_*DRT, k*_ and the reconstructed MS trend *s*_*k*_ for the kth sample paths across all samples of all subjects for each target position. The green vertical line and the red horizontal line represent the mean value of μ_*DRT, k*_ and *s*_*k*_, respectively. The dotted black line is the complete balanced condition defined by Equation (8).

The linear trend of DRT quantified by the slope of the least-squares linear regression line μ_*DRT, k*_ and the linear trend of MS quantified by *s*_*k*_ defined by Equation (7) for the *k*th sample path were compared by the scatter plot for each target position across all sampling path from all subjects (Figure 9, in which the plotted points represent single sampling paths across all subjects). Negative correlations between them were apparent for all target positions. In addition, they were distributed along the line given by Equation (8) that is satisfied when the centripetal DRT and centrifugal MS are completely counterbalanced.

The vertical green line and the horizontal red line in each panel of Figure 9 represent the mean values $\mu_{DRT}$ and *s* across all subjects. The green line $\mu_{DRT}$ shifted more to the right (positive direction) as the target was displayed more to the left (negative direction), and they shifted more to the left (negative direction) as the target was displayed more to the right (positive direction), which was consistent with the lower middle panel of Figure 4. The red line *s* shifted more downward (negative direction) as the target was displayed more to the left (negative direction), and they shifted more upward (positive direction) as the target was displayed more to the right (positive direction), which was consistent with Figure 4, lower right panel and Figure 8, upper panel.

## Discussion

### Summary

The GZ time series was decomposed into DRT and MS time series. The DRT and MS time series showed unidirectional linear trends in opposite directions. The slope (i.e., the velocity) of the linear trends altered depending on the horizontal position of the fixation target. DRT during inter-MS intervals tended to migrate toward the front-facing position, and MS pulled back the GZ position toward the fixation target intermittently. MSD analysis of the GZ and DRT time series clarified that the GZ time series (FEM) behaved like Brownian motion on the short-time scale (0.002–0.1 s) and exhibited antipersistence on the long-time scale (0.2–10 s), whereas the DRT time series was similar to the Brownian motion over the entire time scale (0.2–10 s). These diffusion characteristics were common for all conditions at the fixation target position, which means that the stochastic property of diffusion in DRT alone, such as positive persistence, might not be the major determinant of the unidirectional linear trend of DRT. In other words, the GZ relaxes very slowly (~0.1°/s) with the centripetal DRT toward the front-facing position during inter-MS intervals caused by a deterministic driving force, as if there always exists a quasi-stable equilibrium posture in the front-facing position.

### Comparison of the Centripetal DRT Trend With Previous Studies

The linear trend (velocity) of the centripetal DRT was at most about 0.1°/s at the L2 and R2 conditions placed at 15.2° leftward and rightward shifted positions, respectively (Figure 4). Centripetal DRT velocity estimated by Bertolini et al. (2013) was about 0.3°/s at the corresponding eccentricity, i.e., for the L2 and R2 conditions, which was three times larger than the velocity estimated by the current study. One of the possible causes of this discrepancy is that Bertolini et al. measured DRT velocity in the transient state, that is, not during persistent GZ fixation but during GZ tracking of a quasi-fixation LED target that flashed briefly for 0.05 s every 2 s and shifted between −40° and 40° synchronously with the flash. Such an experimental setting might affect DRT velocity because the GZ in this case might not be able to settle to its steady state within the short time of 0.05 s.

### Random-Walk Analysis

The unit slope of the centripetal DRT time series in the Brownian motion analysis (diffusion plot) suggests that the linear trend of the centripetal DRT is not caused by positive persistence of the DRT time series for the following reason, which can be clarified as follows. First, let us consider a random process with positive persistence, i.e., an increment of the process is followed by an increment in the same direction as the previous increment with probability greater than 1/2. Due to this property of positive persistence, the process with positive persistence tends to exhibit a drift-like behavior in one direction. Note that a process with positive persistence exhibits a Hurst exponent *H* > 1/2 and the scaling exponent (a slope of the diffusion plot) 2*H* > 1. On the other hand, Brownian motion is neutral in terms of persistence. Namely, an increment of the Brownian motion is independent of the successive increment, and its scaling exponent (a slope of the diffusion plot) is 1, as in the DRT time series. Based on this consideration, we can conclude that the linear trend in the centripetal DRT is not caused by positive persistence of the DRT time series. This result suggests that the linear trend in the DRT time series is caused by a deterministic drifting component. Indeed, if we consider a random process *X*(*t*)≡*t*+*B*(*t*), where *t* is a linear trend proportional to time and *B*(*t*) is the Brownian motion, it can be confirmed that *X*(*t*) exhibits a linear trend and the scaling exponent (the slope of the diffusion plot) is 1.

The diffusion property of the GZ time series characterized by the MSD analysis showed a crossover phenomenon at the critical time lag around 0.1–0.2 s that separates the Brownian-motion-like behavior in the short-time scale and antipersistence on the long-time scale, regardless of the horizontal eccentricities (Figure 5). As the mean MS frequency was about 2 Hz, the Brownian-motion-like behavior of the GZ time series on the short-time scale must be dominated by that of the DRT time series. Indeed, the MSD of the GZ time series was similar to that of the DRT time series on the short-time period (0.002–0.1 s). As the scaling exponent (2*H*) of the DRT time series was close to 1 for both short- and long-time scales, we could conclude that the diffusion property of DRT can be modeled by the Brownian motion over the entire time period (0.002–10 s). On the other hand, the antipersistence with the scaling exponent less than 1 of the GZ time series on the long-time scale (0.2–10 s) might be caused by MS that counteracted the diffusion of DRT.

The result of our random-walk analysis was qualitatively consistent with the pioneering research by Engbert and Kliegl (2004), but quantitatively different. In their study, the critical point of FEM appeared around 0.02–0.04 s, whereas the critical point of this study was located at around 0.1–0.2 s in this study, i.e., about five times larger for the current study. Possible reasons for the difference could be the dimension and length of FEM of interest. They applied the random-walk analysis to a two-dimensional FEM measured for 3 s, whereas we used a one-dimensional (horizontal) FEM recorded up to 30 s with a typical length of *T*_*GZ*_~16 s for the GZ time series. Thus, direct comparisons between our results and their study are less meaningful. However, the diffusion properties of FEM may change according to the spatial and temporal size of interest. Nevertheless, as discussed above, because the critical time lag (crossover point) of the MSD analysis of FEM must be closely related to the occurrence timing and amplitude of MS, the crossover point of our estimate might be more plausible from a mechanistic viewpoint.

### FEM as GEN With More Randomness and a Slower Centripetal DRT Trend Compared to EPN

This study revealed that alternating repetitions between centripetal drifts and centrifugal saccades as in physiological GEN, also referred to as EPN during FEM with large horizontal eccentricities, even during FEM with small horizontal eccentricities, but with slower centripetal DRT and more stochasticity in the oscillation period. GEN stochasticity during FEM can be characterized by the SD of the inter-(micro)saccadic intervals. In this study, both the mean and SD of the leftward and the rightward IMSI were the inverse of the MS rate for the Poisson-point-process, which were about 1.25 s. In addition to this randomness, the major cause of GEN stochasticity during FEM was that MS occurred not only in the centrifugal direction against centripetal DRT, but also in the centripetal direction, which lowered the periodicity of GEN during FEM. On the other hand, EPN is much more periodic because MS-like jumps apparently occurred only in the centrifugal direction in EPN (Abel et al., 1978; Shallo-Hoffmann et al., 1990) although the SD of the oscillation period for EPN (Shallo-Hoffmann et al., 1990) is not much different from the SD of the IMSI during FEM. In this way, the stochasticity of the GEN-like behavior during FEM was much larger than EPN with large eccentricities. This finding is novel, which might contribute to a deeper understanding of both physiological GEN and FEM. The stochasticity of GEN can be argued as follows. In front-looking, MS drivers are influenced by a variety of factors, such as proper foveating, and peripheral attention. On the other hand, when looking far to the side, all of these factors are trumped by a bigger force favoring to relax the eye muscles back to the primary position (resulting in the centripetal drift followed by corrective centrifugal saccades). In other words, the properties of FEM may not be fully stochastic and are influenced by a variety of task demands; a demand of eccentric GZ fixation might add an extra factor of the eye relaxing back to its primary position, which might appear it somehow “reduces” stochasticity.

The time constant of the exponential centripetal DRT can be defined as the quotient of the difference between the target position and the equilibrium position divided by a DRT trend, which characterizes the leakiness of the oculomotor velocity-to-position neural integrator (Kheradmand and Zee, 2011). If the equilibrium point is assumed to be at the horizontal center of 0°, the time constant obtained from this study was very slow, which was about 250 s if calculated from the mean DRT trend, and about 85 s even in the smallest case. These time constants are much larger than the time constant of a DRT trend in the periodic EPN, and also larger than the time constant of the centripetal drifts in the previous studies (Becker and Klein, 1973; Robinson et al., 1984; Eizenman et al., 1990; Reschke et al., 2006). Particularly, in previous studies, subjects were required to memorize and fixate continuously on the position of the fixation target presented instantaneously in complete darkness, but in the present study, fixation target position was presented continuously in the presence of display light. To the best of our knowledge, this is the first study to show the existence of the slow centripetal DRT in FEM at small target angles other than in complete darkness with large eccentricities.

### Possible Mechanisms of the Centrifugal MS Trend Generation

Linear regression analysis of the MS time series revealed that the centrifugal MS trend counterbalanced the centripetal DRT trend (Figure 4), and the MS trend was generated by the target position-dependent small modulation of the total MS amplitudes (Table 1) as well as the target position- and jump direction-dependent small modulation in the occurrence frequency of MS events (Figure 8), albeit without statistical significance in these modulations. Despite the absence of significant position-dependent differences in each of the rightward and leftward MS amplitudes, a significant dependence of the target position on the mean total MS amplitude, which averages the MS amplitudes without distinction between rightward and leftward jumps, implies a non-negligible dependence of the target position on MS amplitude for each of the rightward and leftward MS.

A detailed analysis of the MS time series and their centrifugal trend was performed based on the four parameters of MS, i.e., ${\hat{\lambda }}_{+},\;{\hat{\lambda }}_{-},\;{ŵ}_{+},$ and ŵ_−_, which represent the subject-wise means of rates and amplitudes of the rightward and the leftward MS events, together with the combination of those as ŝ, which is the slope of the centrifugal trend of MS (Figure 8). Such an analysis revealed the complex mechanism of how the centrifugal MS trend is generated. None of the four parameters did not show significant dependence on the target positions, while ŝ reconstructed from the four parameters explained the dependence of the target position on the velocity of the centrifugal trend (Figure 8, top panel). This result implies that MS amplitude and frequency were co-modulated (though very slightly for each of them own) to generate the target position-dependent slope of the centrifugal trend of MS, which counterbalanced the centripetal DRT trend. It has been known that the occurrence frequency of MS is suppressed when subjects concentrate on the fixation intentionally (Winterson and Collewun, 1976; Bridgeman and Palca, 1980). A typical example of such an amplitude modulation occurs when subjects are asked to perform a macroscopic voluntary saccade to track a sudden change in the target position. In this case, the MS amplitude during GZ fixation becomes small prior to the onset of the voluntary saccade, suggesting that MS and voluntary saccades may share a common neural control mechanism. Recent studies have begun to elucidate a detailed MS generation and modulation mechanism by a cortical neural network (e.g., frontal eye field and primary visual cortex) and a subcortical neural network (e.g., superior colliculus and cerebellum), which adapt MS dynamics to a varied situation (Hafed et al., 2009, 2021; Hafed, 2011; Otero-Millan et al., 2011; Arnstein et al., 2015; Peel et al., 2016; Willeke et al., 2019; Buonocore et al., 2021). Such functional neuroanatomy could provide a mechanistic basis of the cooperative control and/or co-modulation of MS amplitude and frequency that were characterized in this study. Establishing a computational model for this mechanism is our future work.

### Target Position-Dependent Counterbalance Between the Linear Trends of DRT and MS

The scatter plot between the linear trends of DRT characterized by μ_*DRT, k*_ and MS characterized by *s*_*k*_ showed that centripetal DRT and centrifugal MS satisfied Equation (8), and the counterbalance point between them (i.e., the point at which the mean linear trends of DRT and MS coincided in the μ_*DRT, k*_-*s*_*k*_ plane) in Figure 9 was shifted from the origin along the line μ_*DRT*_ = −*s* of Equation (8), depending on the target position. This target position-dependent shift of the counterbalance point along the line of Equation (8) explains the GZ control mechanism during GZ fixation with eccentricities because the GZ fixation might lose its stability if the counterbalance point between DRT and MS was shifted away from the line of Equation (8). Interestingly, it seems that eye blinks might reset the GZ position to the desired target position when the counterbalance between DRT and MS was shifted away from the line of Equation (8) based on a visual inspection of the upper middle and right panels of Figure 4, where the DRT and/or the MS series tended to be terminated by eye blink, making the length of those sample paths shorter than the others.

### Oculomotor Control Perspective

Fixation on a target shifted to the left or right from the center position is an attention-demanding motor task. Therefore, it is expected that the motor center and motor neurons need to maintain the tonic balance of the tension between the medial and lateral rectus muscles of each eyeball. The existence of migration trends in DRT implies that the tonic control of these antagonist muscles is lost intermittently, and the GZ diffuses away from the fixation target position during “resting” intervals. Furthermore, in this situation, it is conceivable that a stable eyeball posture appears transiently somewhere in the front-facing-side, toward which the eyeball posture may be relaxed, leading to the generation of centripetal DRT. From the viewpoint of motor control, when one fixates on a target position deviated from the center to the left or right, his/her eyeballs are always drawn to their most stable postures by the DRT, which is internal noise, and the errors that increase continuously during the DRT interval are intermittently and stochastically reduced by MS reflecting the control input for correction. This is consistent with existing research with respect to the role of MS in improving fixation stability (Cornsweet, 1956; Engbert and Kliegl, 2004; Ko et al., 2010).

The intermittent loss of tonic balance between antagonist muscles could be due to neuronal fatigue or more strategic optimization, such as to minimize energy expenditure or to increase flexibility in preparation for oculomotor demands.

## Data Availability Statement

The raw data supporting the conclusions of this article will be made available by the authors, without undue reservation.

## Ethics Statement

The studies involving human participants were reviewed and approved by the Ethical Committee of the Graduate School of Engineering Science at Osaka University. The patients/participants provided their written informed consent to participate in this study.

## Author Contributions

MO and TN conceived, designed the research, and interpreted the results of the experiments. MO performed the experiments, analyzed the data and drafted this manuscript. YS and TN advised on the analytical methodologies. MO and YS prepared the figures. TN edited and revised this manuscript. MO, YS, and TN approved the final version of this manuscript.

## Funding

This study was supported by the following grants from the Ministry of Education, Culture, Sports, Science and Technology (MEXT)/Japan Society for the Promotion of Science (JSPS) KAKENHI: 20H05470 (TN), 26242041 (TN), and 20J13713 (MO). MO was supported in part by Program for Leading Graduate Schools of MEXT and the scholarship for human resources development granted by Sumitomo Chemical Co., Ltd., Japan.

## Conflict of Interest

The authors declare that the research was conducted in the absence of any commercial or financial relationships that could be construed as a potential conflict of interest.

## Publisher's Note

All claims expressed in this article are solely those of the authors and do not necessarily represent those of their affiliated organizations, or those of the publisher, the editors and the reviewers. Any product that may be evaluated in this article, or claim that may be made by its manufacturer, is not guaranteed or endorsed by the publisher.
