# Supplementary material for: Stochastic Physiological Gaze-Evoked Nystagmus With Slow Centripetal Drift During Fixational Eye Movements at Small Gaze Eccentricities
Source: Front Hum Neurosci. 2022 May 12;16:842883. doi: 10.3389/fnhum.2022.842883 (PMC9133340; doi:10.3389/fnhum.2022.842883)
Supplement: Supplementary file 1 [file Data_Sheet_1.PDF]

Supplementary Material

Decomposing GZ time series into DRT and MS series

First, for a given raw-GZ time series $X_{rGZ}(t)$, its increment time series $\Delta X_{rGZ}(t)$ is defined as

$$\Delta X_{rGZ}(t) \equiv X_{rGZ}(t) - X_{rGZ}(t-1). \qquad \text{(S1)}$$

By definition, a value of $X_{rGZ}(t)$ at time t can be reconstructed by summing up (integrating) a sequence of $\Delta X_{rGZ}(i)$ from its initial value $X_{rGZ}(0)$ as

$$X_{rGZ}(t) = X_{rGZ}(0) + \sum_{i=1}^{t} \Delta X_{rGZ}(i). \qquad \text{(S2)}$$

Second, we consider a decomposition of $X_{rGZ}(t)$ with its DRT components $X_{DRT}(t)$ and raw-MS components $X_{rMS}(t)$. Later, we define a simplified MS time series $X_{MS}(t)$ by eliminating overshoot components as well as a ballistic transient component of each MS from the raw-MS time series $X_{rMS}(t)$ (Ozawa and Nomura, 2019). Let T_{rGZ} be the whole time interval (an inter-blink interval), on which $X_{rGZ}(t)$ is defined for $t \cdot \delta_s \subset T_{rGZ}$. Similarly, let T_{DRT} and T_{rMS} be the sets of time interval, for which we define

$$\Delta X_{DRT}(t) \equiv \begin{cases} \Delta X_{rGZ}(t), & t \cdot \delta_s \subset T_{DRT} \\ 0, & t \cdot \delta_s \subset T_{rMS} \end{cases} \qquad \text{(S3)}$$

$$\Delta X_{rMS}(t) \equiv \begin{cases} 0, & t \cdot \delta_s \subset T_{DRT} \\ \Delta X_{rGZ}(t), & t \cdot \delta_s \subset T_{rMS} \end{cases} \qquad \text{(S4)}$$

Note that

$$T_{rGZ} = T_{DRT} \cup T_{rMS}, \qquad \text{(S5)}$$

$$T_{DRT} \cap T_{rMS} = \phi. \qquad \text{(S6)}$$

As in Eq. (S2), for given initial values of $X_{DRT}(t)$ and $X_{rMS}(t)$, $X_{DRT}(t)$ and $X_{rMS}(t)$ can be reconstructed from $\Delta X_{DRT}(t)$ and $\Delta X_{rMS}(t)$, respectively. That is, assuming these initial values are equal to the initial value $X_{GZ}(0)$ as

$$X_{DRT}(0) = X_{rMS}(0) = X_{rGZ}(0), \qquad \text{(S7)}$$

we have

$$X_{DRT}(t) = X_{DRT}(0) + \sum_{i=1}^{t} \Delta X_{DRT}(i), \qquad \text{(S8)}$$

$$X_{rMS}(t) = X_{rMS}(0) + \sum_{i=1}^{t} \Delta X_{rMS}(i). \qquad \text{(S9)}$$

Moreover, by taking the sum of Eqs. (3) and (4), we have

$$\Delta X_{DRT}(t) + \Delta X_{rMS}(t) = \begin{cases} \Delta X_{rGZ}(t), & t \cdot \delta_s \subset T_{DRT} \\ \Delta X_{rGZ}(t), & t \cdot \delta_s \subset T_{rMS} \end{cases} \qquad \text{(S10)}$$

Thus, from Eq. (S5), we have

$$\Delta X_{DRT}(t) + \Delta X_{rMS}(t) = \Delta X_{rGZ}(t) \qquad \text{(S11)}$$

for any $t \cdot \delta_s \subset T_{rGZ}$. By taking the sum of Eqs. (8) and (9), we also have

$$X_{DRT}(t) + X_{rMS}(t) = X_{rGZ}(t) + X_{rGZ}(0) \qquad \text{(S12)}$$

or

$$X_{rGZ}(t) = X_{DRT}(t) + X_{rMS}(t) - X_{rGZ}(0) \qquad \text{(S13)}$$

for any $t \cdot \delta_s \subset T_{rGZ}$. Eq. (S13) indicates that we can reconstruct the corresponding time series $X_{rGZ}(t)$ from the decomposed components $X_{DRT}(t)$ and $X_{rMS}(t)$ with an initial value $X_{rGZ}(0)$.

We defined a simplified MS time series $X_{MS}(t)$, simply referred to as the MS time series in this sequel, by eliminating overshoot and transient ballistic components of MS from $X_{rMS}(t)$. $X_{MS}(t)$ was defined for $t \cdot \delta_s \subset T_{MS}$, where T_{MS} was the set of time intervals defined by eliminating the time intervals for the overshoot and ballistic transient components from T_{rMS}. By using $X_{MS}(t)$, we defined a simplified GZ time series $X_{GZ}(t)$, simply referred to as the GZ time series in this sequel. As in Eq. (S13), we have

$$X_{GZ}(t) = X_{DRT}(t) + X_{MS}(t) - X_{GZ}(0) \qquad \text{(S14)}$$

for any $t \cdot \delta_s \subset T_{GZ}$, where

$$T_{GZ} = T_{DRT} \cup T_{MS}. \qquad \text{(S15)}$$

Main sequence

A linear relationship between the maximum amplitude and peak velocity of microsaccades (MS) is called a main sequence, which is often used to verify the correctness of the MS detection. To obtain plausible peak velocity, smoothed velocity defined as weighted moving average over five points is

typically used in the E&K method (Engbert, 2006). For a given two-dimensional gaze position vector $\vec{x}(t) = (x(t), y(t))$, the smoothed velocity is defined as

$$\vec{v}(t) = \frac{\vec{x}(t+2\Delta t) + \vec{x}(t+\Delta t) - \vec{x}(t-\Delta t) - \vec{x}(t-2\Delta t)}{6\Delta t},$$

where Δt is a sampling interval. The peak velocity v_p was obtained as the maximum L^2-norm of the velocity vector during MS

$$v_p = \max(\|\vec{v}(t)\|) = \max\left(\sqrt{v_x^2(t) + v_y^2(t)}\right), \qquad (t_{on} \leq t \leq t_{off}),$$

where $v_x, v_y, t_{on}, and\ t_{off}$ is a horizontal and vertical velocity of $\vec{v}$, and an onset or offset timing of any microsaccade, respectively.

The maximus amplitude a_m was calculated as the maximum L^2-norm of the position vector during MS:

$$a_m = \max(\|\vec{x}(t_{on}) - \vec{x}(t_i)\|) \ \ (t_{on} < t_i \leq t_{off}).$$

The main sequence calculated from the raw-MS time series data for all sample paths across all subjects obtained in this study was drawn using above equations in Fig. S1. Each blue dot in Fig. S1 represents a single MS event. The correlation coefficient between $\log(a_m)$ and $\log(v_p)$ was 0.93.

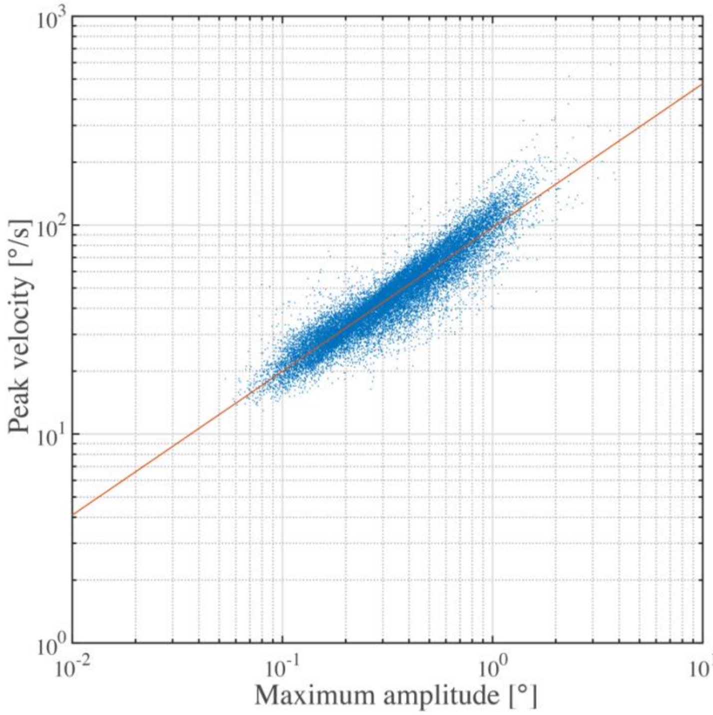

Figure. S1. The main sequence of MS events detected from the raw MS time series data for all sample paths across all subjects. Blue dots are scattered pairs of maximum amplitude and peak velocity, and the orange line is the regression line.
